# Lasing Effect in Symmetrical van der Waals Heterostructured Metasurfaces Due to Lattice-Induced Multipole Coupling

**DOI:** 10.1021/acs.nanolett.3c03522

**Published:** 2023-11-29

**Authors:** Alexei V. Prokhorov, Mikhail Yu. Gubin, Alexander V. Shesterikov, Aleksey V. Arsenin, Valentyn S. Volkov, Andrey B. Evlyukhin

**Affiliations:** † Emerging Technologies Research Center, XPANCEO, Dubai 00000, United Arab Emirates; ‡ Institute of Quantum Optics, Leibniz Universität Hannover, Hannover 30167, Germany

**Keywords:** van der Waals heterostructured metasurfaces, lasing effect, Mie resonances, octupole quasi-trapped modes, laser rate equations

## Abstract

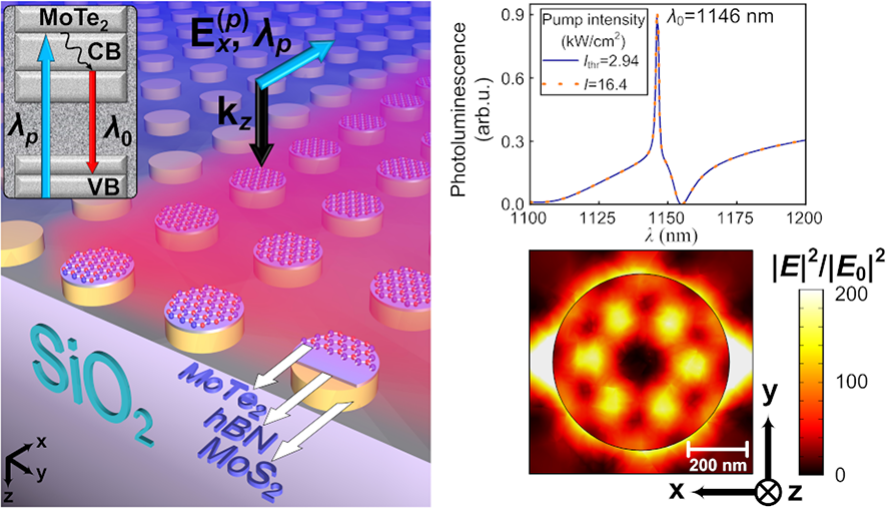

New practical ways to reach the lasing effect in symmetrical metasurfaces have been developed and theoretically demonstrated. Our approach is based on excitation of the resonance of an octupole quasi-trapped mode (OQTM) in heterostructured symmetrical metasurfaces composed of monolithic disk-shaped van der Waals meta-atoms featured by thin photoluminescent layers and placed on a substrate. We revealed that the coincidence of the photoluminescence spectrum maximum of these layers with the wavelength of high-quality OQTM resonance leads to the lasing effect. Based on the solution of laser rate equations and direct full-wave simulation, it was shown that lasing is normally oriented to the metasurface plane and occurs from the entire area of metasurface consisting of MoS_2_/hBN/MoTe_2_ disks with line width of generated emission of only about 1.4 nm near the wavelength 1140 nm. This opens up new practical possibilities for creating surface emitting laser devices in subwavelength material systems.

Dielectric nanophotonics has come a long way in a very short time from the first steps in understanding the general principles of excitation of resonances in dielectric nanoparticles^1−3^ to the practical creation of nanoantennas,^4^ subwavelength waveguides,^5^ nonlinear nanoconverters of radiation,^6^ biosensors,^7^ and nanolasers.^8,9^ A promising direction of nanophotonics is associated with the use of a class of layered van der Waals (vdW) materials, whose prominent representatives are transition-metal dichalcogenides (TMDs). They have a unique combination of high refractive index,^10^ strong optical anisotropy,^11,12^ very pronounced exciton resonances,^13−15^ and nonlinear properties.^16^ All this has already allowed scientists to use such materials for light conversion by its scattering on multilayer TMD nanoresonators and their lattices^17−19^ as well as by achieving the strong coupling conditions in the process of inelastic light-matter interaction^10,20−22^ and realization of lasing in such materials.^23,24^ At the same time, the rapid progress of recent years in the area of material science for two-dimensional materials^25,26^ allowed researchers to take a considerably fresh look at the problem of tuning of optical properties of such materials. In particular, the strategy of combining monolayers or thin films of vdW materials into stacks^27−31^ allows the creation of heterostructures with a very wide range of optical properties. The design and fabrication of light-emitting devices, including diodes and lasers based on vdW heterostructures,^32−35^ are of particular interest here. One of the promising approaches for these purposes is the combination of vdW heterostructures and metasurfaces, used as resonators, for the amplification of photoluminescence and increasing of quantum yield, which opens up new possibilities for modern nanophotonics.^36^

In the first works devoted to vdW lasers, the direct bandgap monolayers of WSe_2_,^37^ MoS_2_,^38^ and MoTe_2_,^39,40^ transferred to resonators with different geometries, were used. More complicated vdW MoS_2_/WSe_2_ heterostructures in the regime of hybridization of exciton resonances have been used in combination with a SiN cavity^41^ and with a photonic crystal cavity (PhCC).^42^ Thus, the combination of vdW heterostructures with resonators opens up new possibilities for creating passive and active ultracompact optical devices. In particular, the integration of TMD layers with Mie-resonant dielectric nanostructures has been used for these purposes.^36^ It should be noted that the introduction of vdW materials into modern photonics is only at the initial stage, and we have the right to expect that further development of this process will lead to new and important practical results.

In particular, in our work, we predict and show that the lasing effect can be obtained in heterostructural metasurfaces made from only vdW materials without the use of other resonant systems. To do this, we propose to combine the high-Q Mie-resonant response of the building blocks of the MoS_2_ metasurface with the photoluminescence of an additional thin active layer of MoTe_2_ placed on the upper side of the building blocks and separated from MoS_2_ by a thin hexagonal boron nitride (hBN) layer. The ability to maintain the high-Q Mie resonances results from the higher refractive index of vdW materials^11,43^ in excess of conventional dielectric photonics materials such as silicon and germanium.^2,44^ Our approach is based on the recently revealed mechanism for the excitation of an electric octupole quasi-trapped mode (OQTM) in symmetrical MoS_2_ metasurfaces.^45^ It was shown that the electric octupole mode, which does not radiate electromagnetic energy to the far-field region from the metasurface, is resonantly excited due to its coupling with electric dipole mode that in turn is excited by an incident light wave. In a homogeneous medium, such as air, the energy transfer between the dipole and octupole modes leads to the appearance of a narrow dip in the spectrum of a wide electric dipole resonance as well as to the implementation of the induced transparency effect with simultaneous excitation of the electric octupole resonance. In this Letter, we propose to use such a metasurface supported octupole resonance as a subdiffractive distributed resonator. By matching the OQTM wavelength and the maximum photoluminescence for the active layer, we tune the pump intensity and carrier density in order to achieve positive feedback and lasing in such a system due to the coupling effect. Our theoretical approach is based on solving the problem of reflection and transmission of external radiation through a two-dimensional array of particles using COMSOL Multiphysics facilities as well as on the numerical solution of rate equations^46^ for the carrier density and photon density of the signal field at a given wavelength in the active medium (detailed discussion of methods and sequence of calculations are presented in Supporting Information).

The proposed system is schematically shown in Figure 1a. We consider the solid disks made of a combination of thick MoS_2_ layer and few-atomic-layer MoTe_2_ material as the building blocks of the metasurface placed on a quartz substrate, see Figure 1a and Figure S2a in Supporting Information. For experimental conditions it could be necessary to use a few-layer hBN between MoS_2_ and MoTe_2_ subsystems to avoid the formation of heterobilayer^42^ and prevent charge transfer between layers. However, for simplicity in numerical simulations, the system can be considered without a few-layer hBN, since its inclusion does not affect the calculated results. Note that suggested metasurfaces could be fabricated by successively stacking flakes of various TMDs^49−51^ followed by applying the electron beam lithography process for the obtained stack.^52^

**Figure 1:**
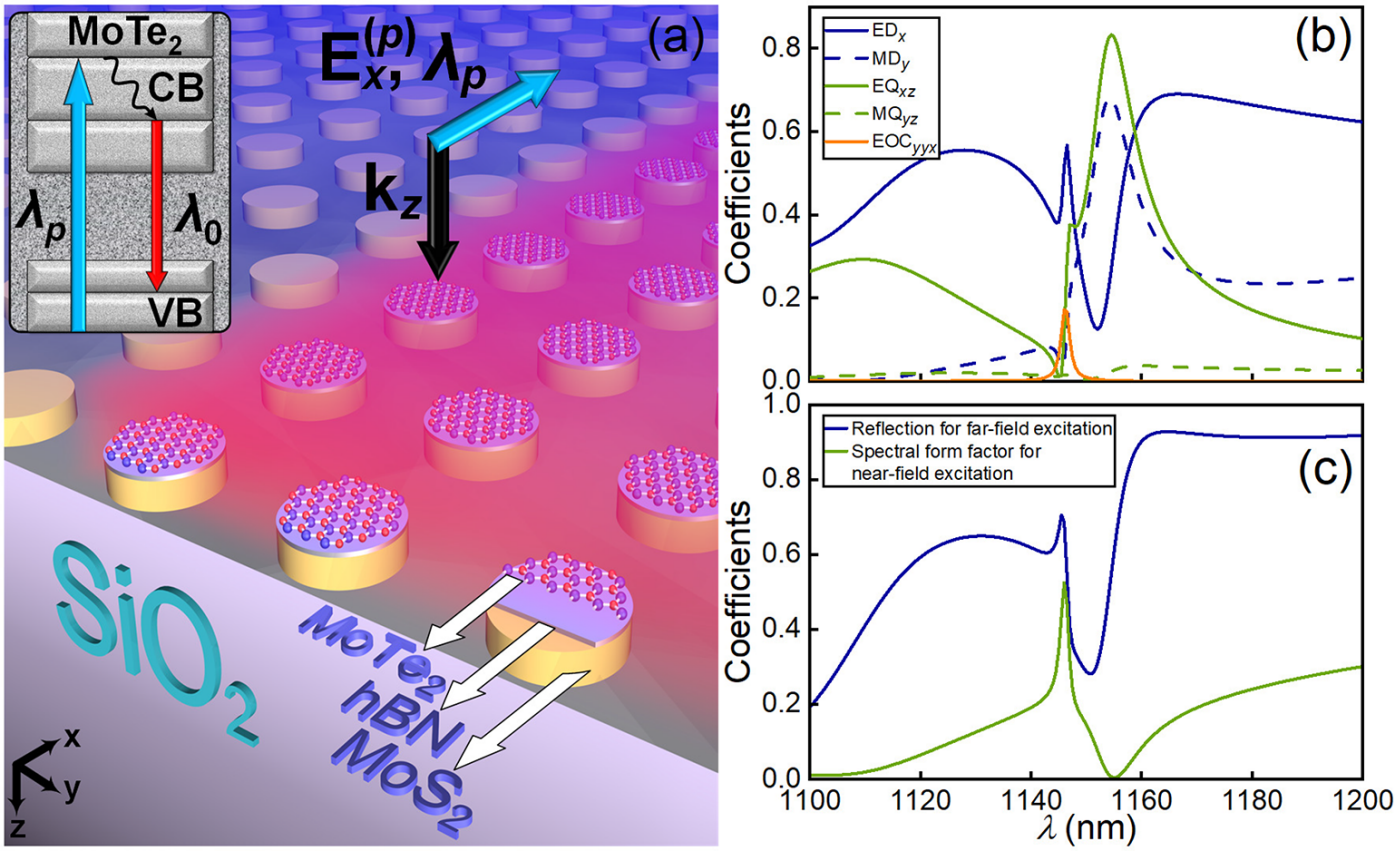
(a) Sketch of the metasurface based on MoS_2_/hBN/MoTe_2_ heterostructure in the OQTM regime. (b) Spectra of the absolute values of the multipole contributions ED_*x*_, MD_*y*_, EQ_*xz*_, MQ_*yz*_ to the reflection coefficient (R) as well as the value EOC_*yyx*_ corresponding to the nonradiating electric octupole moment (EOC) of the MoS_2_/hBN/MoTe_2_ disk with radius *R* = 300 nm and height *H* = 233 nm from the metasurface with period *P* = 760 nm placed on SiO_2_ substrate and irradiated by *E*_*x*_(*k*_*z*_) wave from the far-field region. (c) Reflection coefficient and resonator spectral form factor for the MoS_2_/hBN/MoTe_2_ metasurface with parameters corresponding to panel (b) and excited by wave *E*_*x*_(*k*_*z*_) from the far-field region (solid blue curve) as well as from the near-field region (solid green curve) by means of pumped MoTe_2_ layers. The designations ED_*x*_, MD_*y*_, EQ_*xz*_, MQ_*yz*_, and EOC_*yyx*_ correspond to the contributions of the electric dipole (ED), magnetic dipole (MD), electric octupole (EQ), magnetic quadrupole (MQ), and electric octupole (EOC), respectively, to the reflection (see refs (45), (47), and (48) and Supporting Information for details).

We carried out the optimization of metasurface composed of MoS_2_ on MoTe_2_ disks similar to ref (45) and found out that the addition of two-layered MoTe_2_ material (MoTe_2_ bilayer) does not destroy the effect of the OQTM excitation (details of the method are presented in Supporting Information). The considered effect of OQTM arises at the wavelength λ_*v*_ = 1138 nm when choosing MoS_2_/hBN/MoTe_2_ disks (material layers oriented parallel to disk’s base) with height *H* = 233 nm (including 1.4 nm thickness of MoTe_2_ placed on the top base of the disk) and radius *R* = 300 nm and period *P* = 760 nm of metasurface placed in air, see the multipole decomposition and spectra of reflection and transmission for OQTM regime shown in Figure S2b,c in Supporting Information. Note that in the considered case, the regime of diffraction into the substrate corresponds to the wavelength range λ < 1070 nm that is outside the spectral range in Figure 1c. The choice of the parameters is due to the need to match the octupole resonance with the interband transition for MoTe_2_ material in the vicinity of wavelength λ_*v*_^53^ as well as the possibility to maintain the subdiffraction regime when the metasurface is placed on the SiO_2_ substrate.^54^

The location of a metasurface on a dielectric substrate is an important distinction with respect to ref (45) and is of fundamental importance for observing lasing in such a system. In particular, the presence of dielectric SiO_2_ substrate does not violate the conditions for excitation of the OQTM resonance due to the coupling mechanism but, at the same time, provides the appearance of an additional way of interparticle interaction through the wave reflected from the dielectric boundary. As a result, in contrast to the case of location in the air, there is a small red shift of the octupole resonance to the wavelength λ_s_ = 1146 nm (compare the octupole resonance position in Figures 1b and S2b in Supporting Information). Besides, an additional peak, which exactly corresponds to the spectral position of the octupole resonance, appears on the profile of the broad dipole resonance, as can be seen from the blue curve in Figure 1b. Moreover, other multipole moments also have spectral features at the octupole resonance, which indicates that the presence of a substrate leads to coupling between all multipoles (as shown in Figure 1b). Formally, strong all-multipole coupling can be associated with the breaking of reverse symmetry due to the presence of the substrate.^55^ As a result, in the considered regime, the energy exchange with the pump field mainly occurs with the mode components ED_*x*_, EQ_*xz*_, and EOC_*yyx*_.

Thus, the location of metasurface on a substrate, on the one hand, makes the system more adapted for experimental realization and, on the other hand, does not lead to the suppression of electric dipole moment and to the induced transparency at the spectral position of the octupole resonance. As a result, a narrow peak associated with resonant coupling between electric dipole and electric octupole modes appears in the reflection spectrum of the metasurface placed on the substrate (see the resonant peak at λ = 1146 nm of the reflection coefficient in Figure 1c calculated for the far-field irradiation conditions). Note that the dip near the wavelength 1150 nm in Figure 1c corresponds to the suppression of backward light radiation due to destructive multipole interference.^56,57^

Next, we analyze the properties of a metasurface (distributed resonator system) using near-field excitation from MoTe_2_ layers of each disk, which is necessary for further analysis of the system’s photoluminescence properties. In this case, instead of the reflection spectrum, we calculate the resonator spectral form factor, which is the spectrum of the total radiation intensity from the metasurface normalized to the intensity of the source (method is discussed in Supporting Information). For the above-mentioned system’s parameters, there is an increase in the concentration of the near field in each disk related to an additional narrowing of the OQTM resonance; see the green curve in Figure 1c. In particular, for the case of near-field pumping as in Figure 1c, the width of the resonance at λ = 1146 corresponding to the OQTM is full width at half-maximum (FWHM) = 1.7 nm (with the quality factor *Q*_0_ = 674).

Having studied the properties of a distributed resonator using far-field and near-field excitation, we proceed to consider the lasing scheme shown in Figure 1a. The MoTe_2_ photoluminescence wavelength λ_0_ is determined by the dependence of the bandgap Δ_g_ on the number of layers.^53^ Two-layer MoTe_2_ material with Δ_g_ = 1.08 eV and photoluminescence peak at the wavelength λ_0_ = λ_s_ = 1146 nm at room temperature^58^ can be used as part of the metasurface. Initially, the parameters of the MoS_2_/hBN/MoTe_2_ metasurface were chosen in such a way that the wavelengths of octupole resonance and photoluminescence λ_0_ in two-layer MoTe_2_ material coincide. In simulation, we used the complex refractive index  = *n* + iα for MoTe_2_, and it takes the value^12^ at the wavelength λ_0_.

We assume that the system is pumped by a He–Ne continuous-wave (CW) laser at the wavelength λ_p_ = 633 nm.^39^ The pump wave *E*_*x*_^(*p*)^(*k*_*z*_) normally irradiates the metasurface, see Figure 1a. As a result of the interband transition and radiative recombination, a strong near field is generated by the MoTe_2_ bilayer with a maximum value at the wavelength λ_0_. Since the octupole eigenmode of a lattice is tuned exactly to this wavelength, positive feedback is realized in the system due to the coupling effect between the near field of MoTe_2_ layer, the electric dipole, and electric octupole modes of the total metasurface. Under these conditions, the metasurface acts as a distributed resonator, which should provide a narrowing of the photoluminescence line and, under certain conditions, lasing from the metasurface.

To describe the laser generation in the system, we use the rate equations for the carrier density *N* and photon density *S* of the signal field in the active area determined by the MoTe_2_ material. Following the approach used for similar systems,^8,46,59^ these equations can be written in the following form
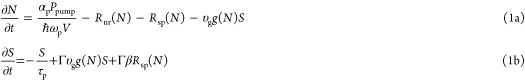
1awhere *V* is the volume of MoTe_2_ bilayer in each disk (*V* = 3.96 × 10^–22^ m^3^), *ℏω*_p_ is the photon energy of external optical pump, α_p_ is the absorption coefficient of MoTe_2_ material for pump wave *E*_*x*_^(*p*)^(*k*_*z*_) at the wavelength λ_p_, τ_p_ = *Q*_0_/ω_0_ is the lifetime of the lasing mode, ω_0_ = 2*πc*/λ_0_ and *Q*_0_ are the frequency and quality factor of the lasing mode, respectively, Γ is the confinement factor of the lasing mode, β is the spontaneous emission factor determined by Purcell factor^60^ (corresponding calculation is presented in S5.4 of Supporting Information), and *P*_pump_ is the pumping power. The quality factor *Q*_0_ is determined by the resonance of the OQTM excited in the metasurafce and extracted from numerical simulation. The following parameters are given in eqs 1: *R*_nr_ = *N*/τ_nr_ + *CN*^3^ and *R*_sp_ = *N*/τ_sp_ are the nonradiative recombination and total spontaneous emission rates, respectively; *g*(*N*) = *a*(*N* – *N*_tr_) is the active medium gain. Here, τ_nr_ and τ_sp_ are the nonradiative and spontaneous recombination lifetimes, respectively, *C* is the Auger recombination coefficient, *a* is the linear gain coefficient, *N*_tr_ is the transparency carrier density, υ_g_ = *c*/*n*_g_ is the material group velocity of the active medium for the lasing mode, and *c* is the light speed in vacuum, *n*_g_ is the group refractive index of the material; we suppose that *n*_g_ = *n*(λ_0_). Next, following ref (61), we choose τ_sp_ = 3 ps and τ_nr_ = 23 ps for the MoTe_2_ as well as *C* = 10^–40^ m^6^ s^–1^,^8^ see Table S1, where the references to the experimental works containing the used parameters and their values for MoTe_2_ are presented.

In the general case, the stationary solutions of the system of nonlinear eqs 1 are cumbersome; therefore, we use the parametric solutions of system (eqs 1) in the stationary case for the values *P*_pump_ and *S*, where the variable parameter is *N*. This approach is reasonable from the applied point of view, if MoTe_2_ is additionally gated by an external field in the regime of varying *N*.^62−66^ Stationary solutions of the system of eqs 1 for the photon density *S*_s_ and pumping power *P*_pump_ depend on the stationary value of the density of charge carriers *N*_s_ and take the following form.^46^
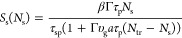
2a
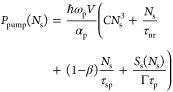
2b

Figure 2a shows the parametric gain curves for *S*_s_ versus pump intensity *I* = *P*_pump_(*N*_s_)/*S*_disk_ corresponding to the MoS_2_/hBN/MoTe_2_ metasurface shown in Figure 1a. Here *S*_disk_ = *πR*^2^ is the area of the active medium (MoTe_2_ material in one building block). Switching on and subsequently increasing the intensity of the pump field, one can determine the threshold of laser generation using the inflection point for gain curve *S*_s_(*I*) of the signal field plotted in double logarithmic scale, which corresponds to the following condition for the second derivative:^39,67^.

**Figure 2:**
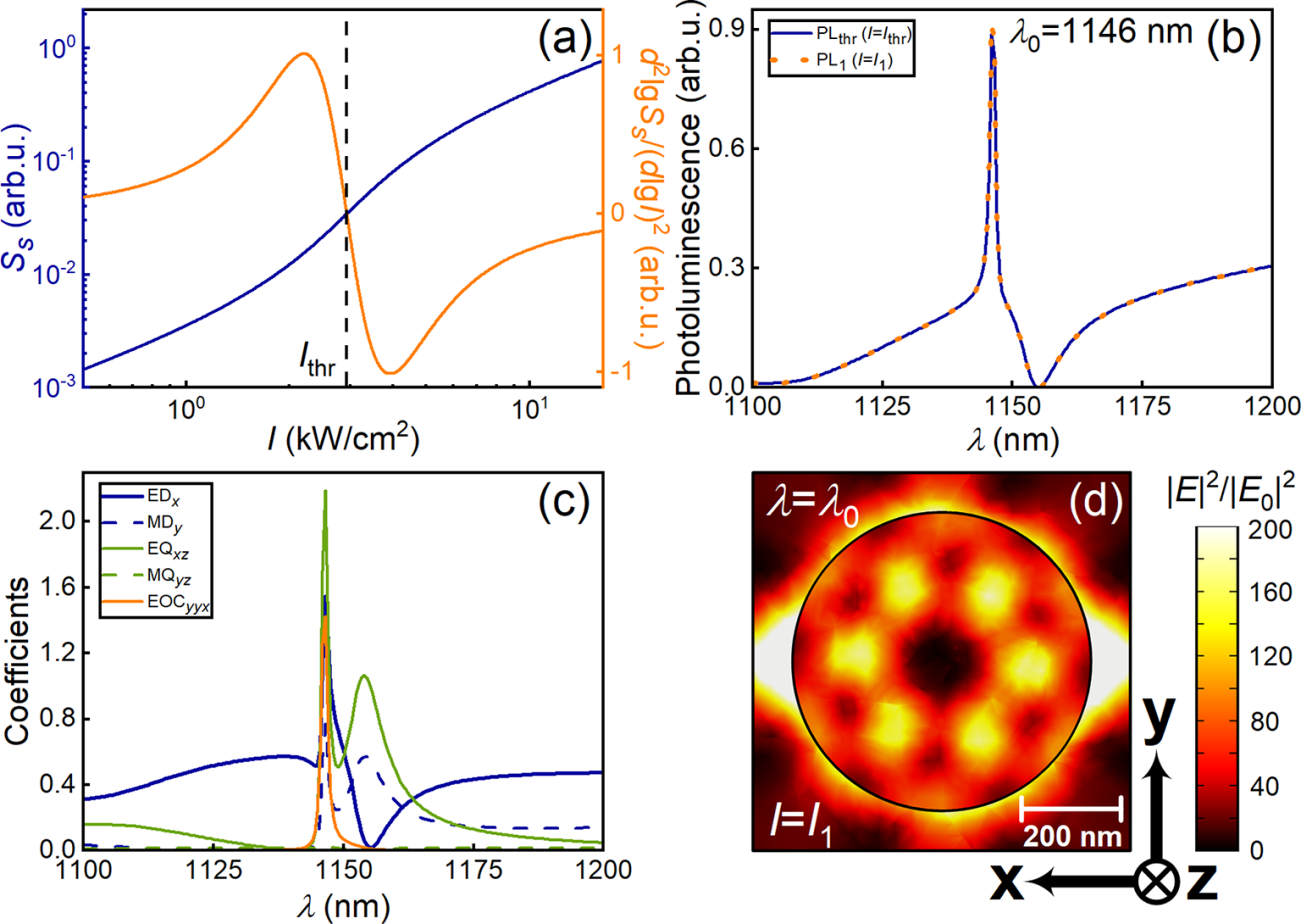
(a) Gain curves for the heterostructured MoS_2_/hBN/MoTe_2_ metasurface with parameters corresponding to Figure 1 at the lasing wavelength λ_0_ = 1146 nm. (b) Photoluminescence spectra (correspond to the normalized intensity of emission from metasurface) for the metasurface shown in Figure 1a for different pump intensities: *I*_thr_ = 2.94 kW/cm^2^ (*k*_g_ = −0.0522), *I*_1_ = 16.4 kW/cm^2^ (*k*_g_ = −0.0821). (c) Spectra of the absolute values of the multipole contributions ED_*x*_, MD_*y*_, EQ_*xz*_, and MQ_*yz*_ to the photoluminescence of metasurface as well as the value EOC_*yyx*_ corresponding to the nonradiating octupole moment of the disks in the metasurface with parameters as in panel (b) under the action of pump field with intensity *I*_thr_. (d) Visualization of the electric field distribution calculated for individual building block of MoS_2_/hBN/MoTe_2_ metasurface in the disk center with parameters as for panel (c).

The threshold conditions for MoS_2_/hBN/MoTe_2_ metasurface are realized for the pump field intensity equal to *I*_thr_ = 2.94 kW/cm^2^ with gain coefficient *g*_thr_ = 5728 cm^–1^ (linear gain coefficient equals to *a* = 6.9 × 10^–14^ cm^2^) and density of charge carriers *N*_thr_ = 2.44 × 10^17^ cm^–3^, see dependencies in Figure 2a, which were obtained using data from Table S2 in Supporting Information.

Photoluminescence (radiation from the system) can be simulated for an optically pumped metasurface using the near-field excitation from MoTe_2_ bilayer of each disk in the regime of varying the imaginary part of MoTe_2_ effective relative permittivity ε_eff_(ω), see the section S5.2 in Supporting Information, where formulas for ε_eff_(ω) takes into account the change in the carrier density *N* according to eqs (1)–(2) under conditions of OQTM-resonator. Figure 2b shows the comparison of numerically calculated photoluminescence spectra of metasurface in the threshold and above-threshold regimes for various values of pump intensity corresponding to the different values of parameter^68,69^ that determines imaginary part of ε_eff_(ω). For the parameters presented in Table S1, the effective relative permittivity of MoTe_2_ for threshold conditions equals to ε_eff_(λ_0_) = 19.3096 – *i*0.4619, where we take into account the change of the real part of relative permittivity near the resonance, see the section S5.3 in Supporting Information. In threshold conditions, the quality factor of the OQTM resonance is *Q* = 819 with FWHM = 1.4 nm, see Figure 2b, and the lifetime of the lasing mode is τ_p_ = 4.1 × 10^–13^ s. The photoluminescence spectrum for threshold and above-threshold conditions is, in fact, determined by the characteristics of the resonator (metasurface) and slightly changes with increasing pump intensity, which is a peculiarity of the considered system. Note that the octupole quasi-trapped mode can be excited in relatively small metasurfaces;^54^ therefore, experimental observation of lasing should also be possible in spatially limited metasurfaces. The multipole analysis in Figure 2c performed for the photoluminescence spectrum in Figure 2b was based on the same definitions of the various multipole contributions to the reflection coefficient as in section S3 of Supporting Information but with the replacement of the scattered fields by the fields emitted from the metasurface. This analysis demonstrates a narrowband resonance for several multipoles at once, which is due to both the dipole-octupole coupling and the action of the substrate; see Figure 2c. Thus, the laser emission is normally oriented to the metasurface plane and has a complex multipole nature. At the same time, a considerable concentration of the electric field occurs in each disk, and its distribution has a characteristic octupole shape in Figure 2d.

Summarizing, thus, the possibility of laser generation from a metasurface based on vdW heterostructures using a new mode selection mechanism based on multipole coupling in the lattice was shown. Note that the considered metasurfaces can be fabricated from a stack assembled from various materials using the electron beam lithography method. A remarkable feature of the considered resonances is the low sensitivity to periodicity inaccuracies and boundary effects for the real metasurface.^54^ Moreover, even with a low level of coherence and the absence of the possibility to control the quantum statistics of photons,^70^ the proposed metasurfaces allow one to observe the partially coherent radiation from the large area in the direction perpendicular to the surface. It is worth noting that wavelength tuning, in this case, can be realized by filling such a system with an electrically controlled optically transparent medium. However, this requires the control of the effective refractive index for the environment in order to avoid the diffraction into the substrate and destruction of the lasing regime. Further development of this work may be related to the study of metasurfaces combined from monolithic MoS_2_ and heterostructured MoS_2_/hBN/MoTe_2_ disks. Optimization of the parameters and excitation conditions in them is necessary to realize coherent on-demand emission from the given disks. Such pixel-lasing metasurfaces may be promising for the creation of next-generation laser displays.

## Supporting Information Available

Additional figures, calculation details, and data for the system parameters (PDF)

## References

[ref1] Evlyukhin A. B.; Reinhardt C.; Seidel A.; Luk’yanchuk B. S.; Chichkov B. N. Optical response features of Si-nanoparticle arrays. Phys. Rev. B 2010, 82, 045404 10.1103/PhysRevB.82.045404.

[ref2] Evlyukhin A. B.; Novikov S. M.; Zywietz U.; Eriksen R. L.; Reinhardt C.; Bozhevolnyi S. I.; Chichkov B. N. Demonstration of Magnetic Dipole Resonances of Dielectric Nanospheres in the Visible Region. Nano Lett. 2012, 12, 3749–3755. 10.1021/nl301594s. 22703443

[ref3] Kuznetsov A. I.; Miroshnichenko A. E.; Fu Y. H.; Zhang J.; Luk’Yanchuk B. Magnetic light. Sci. Rep. 2012, 2, 492 10.1038/srep00492. 22768382 PMC3389365

[ref4] Krasnok A. E.; Miroshnichenko A. E.; Belov P. A.; Kivshar Y. S. All-dielectric optical nanoantennas. Opt. Express 2012, 20, 20599–20604. 10.1364/OE.20.020599. 23037107

[ref5] Bakker R. M.; Yu Y. F.; Paniagua-Domínguez R.; Luk’yanchuk B.; Kuznetsov A. I. Resonant Light Guiding Along a Chain of Silicon Nanoparticles. Nano Lett. 2017, 17, 3458–3464. 10.1021/acs.nanolett.7b00381. 28463510

[ref6] Shcherbakov M. R.; Neshev D. N.; Hopkins B.; Shorokhov A. S.; Staude I.; Melik-Gaykazyan E. V.; Decker M.; Ezhov A. A.; Miroshnichenko A. E.; Brener I.; Fedyanin A. A.; Kivshar Y. S. Enhanced Third-Harmonic Generation in Silicon Nanoparticles Driven by Magnetic Response. Nano Lett. 2014, 14, 6488–6492. 10.1021/nl503029j. 25322350

[ref7] Yavas O.; Svedendahl M.; Dobosz P.; Sanz V.; Quidant R. On-a-chip Biosensing Based on All-Dielectric Nanoresonators. Nano Lett. 2017, 17, 4421–4426. 10.1021/acs.nanolett.7b01518. 28616986

[ref8] Tiguntseva E.; Koshelev K.; Furasova A.; Tonkaev P.; Mikhailovskii V.; Ushakova E. V.; Baranov D. G.; Shegai T.; Zakhidov A. A.; Kivshar Y.; Makarov S. V. Room-Temperature Lasing from Mie-Resonant Non-Plasmonic Nanoparticles. ACS Nano 2020, 14, 8149–8156. 10.1021/acsnano.0c01468. 32484650

[ref9] Lepeshov S.; Vyshnevyy A.; Krasnok A. Switchable dual-mode nanolaser: mastering emission and invisibility through phase transition materials. Nanophotonics 2023, 12, 3729–3736. 10.1515/nanoph-2023-0249. PMC11636156 39678474

[ref10] Verre R.; Baranov D. G.; Munkhbat B.; Cuadra J.; Käll M.; Shegai T. Transition metal dichalcogenide nanodisks as high-index dielectric Mie nanoresonators. Nat. Nanotechnol. 2019, 14, 679–683. 10.1038/s41565-019-0442-x. 31061517

[ref11] Ermolaev G. A.; et al. Giant optical anisotropy in transition metal dichalcogenides for next-generation photonics. Nat. Commun. 2021, 12, 854 10.1038/s41467-021-21139-x. 33558559 PMC7870936

[ref12] Munkhbat B.; Wróbel P.; Antosiewicz T. J.; Shegai T. O. Optical Constants of Several Multilayer Transition Metal Dichalcogenides Measured by Spectroscopic Ellipsometry in the 300–1700 nm Range: High Index, Anisotropy, and Hyperbolicity. ACS Photonics 2022, 9, 2398–2407. 10.1021/acsphotonics.2c00433. 35880067 PMC9306003

[ref13] Arora A.; Koperski M.; Nogajewski K.; Marcus J.; Faugeras C.; Potemski M. Excitonic resonances in thin films of WSe_2_: from monolayer to bulk material. Nanoscale 2015, 7, 10421–10429. 10.1039/C5NR01536G. 25998778

[ref14] Arora A.; Deilmann T.; Marauhn P.; Drüppel M.; Schneider R.; Molas M. R.; Vaclavkova D.; Michaelis de Vasconcellos S.; Rohlfing M.; Potemski M.; Bratschitsch R. Valley-contrasting optics of interlayer excitons in Mo- and W-based bulk transition metal dichalcogenides. Nanoscale 2018, 10, 15571–15577. 10.1039/C8NR03764G. 30090905

[ref15] Popkova A. A.; Antropov I. M.; Tselikov G. I.; Ermolaev G. A.; Ozerov I.; Kirtaev R. V.; Novikov S. M.; Evlyukhin A. B.; Arsenin A. V.; Bessonov V. O.; Volkov V. S.; Fedyanin A. A. Nonlinear Exciton-Mie Coupling in Transition Metal Dichalcogenide Nanoresonators. Laser Photonics Rev. 2022, 16, 2100604 10.1002/lpor.202100604.

[ref16] Bikorimana S.; Lama P.; Walser A.; Dorsinville R.; Anghel S.; Mitioglu A.; Micu A.; Kulyuk L. Nonlinear optical responses in two-dimensional transition metal dichalcogenide multilayer: WS_2_, WSe_2_, MoS_2_ and Mo_0.5_W_0.5_S_2_. Opt. Express 2016, 24, 20685–20695. 10.1364/OE.24.020685. 27857426

[ref17] Prokhorov A. V.; Shesterikov A. V.; Gubin M. Y.; Volkov V. S.; Evlyukhin A. B. Quasitrapped modes in metasurfaces of anisotropic MoS_2_ nanoparticles for absorption and polarization control in the telecom wavelength range. Phys. Rev. B 2022, 106, 035412 10.1103/PhysRevB.106.035412.

[ref18] Nauman M.; Yan J.; de Ceglia D.; Rahmani M.; Zangeneh Kamali K.; De Angelis C.; Miroshnichenko A. E.; Lu Y.; Neshev D. N. Tunable unidirectional nonlinear emission from transition-metal-dichalcogenide metasurfaces. Nat. Commun. 2021, 12, 5597 10.1038/s41467-021-25717-x. 34552076 PMC8458373

[ref19] Koshelev K.; Tang Y.; Li K.; Choi D.-Y.; Li G.; Kivshar Y. Nonlinear Metasurfaces Governed by Bound States in the Continuum. ACS Photonics 2019, 6, 1639–1644. 10.1021/acsphotonics.9b00700.

[ref20] Schneider C.; Glazov M. M.; Korn T.; Höfling S.; Urbaszek B. Two-dimensional semiconductors in the regime of strong light-matter coupling. Nat. Commun. 2018, 9, 2695 10.1038/s41467-018-04866-6. 30002368 PMC6043564

[ref21] Munkhbat B.; Baranov D. G.; Stührenberg M.; Wersäll M.; Bisht A.; Shegai T. Self-Hybridized Exciton-Polaritons in Multilayers of Transition Metal Dichalcogenides for Efficient Light Absorption. ACS Photonics 2019, 6, 139–147. 10.1021/acsphotonics.8b01194.

[ref22] Qin M.; Duan J.; Xiao S.; Liu W.; Yu T.; Wang T.; Liao Q. Manipulating strong coupling between exciton and quasibound states in the continuum resonance. Phys. Rev. B 2022, 105, 195425 10.1103/PhysRevB.105.195425.

[ref23] Wen W.; Wu L.; Yu T. Excitonic Lasers in Atomically Thin 2D Semiconductors. ACS Materials Lett. 2020, 2, 1328–1342. 10.1021/acsmaterialslett.0c00277.

[ref24] Huang L.; Krasnok A.; Alú A.; Yu Y.; Neshev D.; Miroshnichenko A. E. Enhanced light-matter interaction in two-dimensional transition metal dichalcogenides. Rep. Prog. Phys. 2022, 85, 046401 10.1088/1361-6633/ac45f9. 34939940

[ref25] Novoselov K. S.; Jiang D.; Schedin F.; Booth T. J.; Khotkevich V. V.; Morozov S. V.; Geim A. K. Two-dimensional atomic crystals. Proc. Natl. Acad. Sci. U.S.A. 2005, 102, 10451–10453. 10.1073/pnas.0502848102. 16027370 PMC1180777

[ref26] Geim A. K.; Novoselov K. S. The rise of graphene. Nat. Mater. 2007, 6, 183–191. 10.1038/nmat1849. 17330084

[ref27] Geim A. K.; Grigorieva I. V. Van der Waals heterostructures. Nature 2013, 499, 419–425. 10.1038/nature12385. 23887427

[ref28] Novoselov K. S.; Mishchenko A.; Carvalho A.; Castro Neto A. H. 2D materials and van der Waals heterostructures. Science 2016, 353, aac9439 10.1126/science.aac9439. 27471306

[ref29] Li M.-Y.; Chen C.-H.; Shi Y.; Li L.-J. Heterostructures based on two-dimensional layered materials and their potential applications. Mater. Today 2016, 19, 322–335. 10.1016/j.mattod.2015.11.003.

[ref30] Cai Z.; Liu B.; Zou X.; Cheng H.-M. Chemical Vapor Deposition Growth and Applications of Two-Dimensional Materials and Their Heterostructures. Chem. Rev. 2018, 118, 6091–6133. 10.1021/acs.chemrev.7b00536. 29384374

[ref31] Jiang X.; Chen F.; Zhao S.; Su W. Recent progress in the CVD growth of 2D vertical heterostructures based on transition-metal dichalcogenides. CrystEngComm 2021, 23, 8239–8254. 10.1039/D1CE01289D.

[ref32] Withers F.; Del Pozo-Zamudio O.; Mishchenko A.; Rooney A. P.; Gholinia A.; Watanabe K.; Taniguchi T.; Haigh S. J.; Geim A. K.; Tartakovskii A. I.; Novoselov K. S. Light-emitting diodes by band-structure engineering in van der Waals heterostructures. Nat. Mater. 2015, 14, 301–306. 10.1038/nmat4205. 25643033

[ref33] Bie Y.-Q.; Grosso G.; Heuck M.; Furchi M. M.; Cao Y.; Zheng J.; Bunandar D.; Navarro-Moratalla E.; Zhou L.; Efetov D. K.; Taniguchi T.; Watanabe K.; Kong J.; Englund D.; Jarillo-Herrero P. A MoTe_2_-based light-emitting diode and photodetector for silicon photonic integrated circuits. Nat. Nanotechnol. 2017, 12, 1124–1129. 10.1038/nnano.2017.209. 29209014

[ref34] Yang X.; et al. A Waveguide-Integrated Two-Dimensional Light-Emitting Diode Based on p-Type WSe_2_/n-Type CdS Nanoribbon Heterojunction. ACS Nano 2022, 16, 4371–4378. 10.1021/acsnano.1c10607. 35191308

[ref35] Khelifa R.; Shan S.; Moilanen A. J.; Taniguchi T.; Watanabe K.; Novotny L. WSe_2_ Light-Emitting Device Coupled to an h-BN Waveguide. ACS Photonics 2023, 10, 1328–1333. 10.1021/acsphotonics.2c01963. 37215323 PMC10197165

[ref36] Mupparapu R.; Bucher T.; Staude I. Integration of two-dimensional transition metal dichalcogenides with Mie-resonant dielectric nanostructures. Adv. Phys.: X 2020, 5, 1734083 10.1080/23746149.2020.1734083.

[ref37] Wu S.; Buckley S.; Schaibley J. R.; Feng L.; Yan J.; Mandrus D. G.; Hatami F.; Yao W.; Vučković J.; Majumdar A.; Xu X. Monolayer semiconductor nanocavity lasers with ultralow thresholds. Nature 2015, 520, 69–72. 10.1038/nature14290. 25778703

[ref38] Salehzadeh O.; Djavid M.; Tran N. H.; Shih I.; Mi Z. Optically Pumped Two-Dimensional MoS_2_ Lasers Operating at Room-Temperature. Nano Lett. 2015, 15, 5302–5306. 10.1021/acs.nanolett.5b01665. 26214363

[ref39] Li Y.; Zhang J.; Huang D.; Sun H.; Fan F.; Feng J.; Wang Z.; Ning C. Z. Room-temperature continuous-wave lasing from monolayer molybdenum ditelluride integrated with a silicon nanobeam cavity. Nat. Nanotechnol. 2017, 12, 987–992. 10.1038/nnano.2017.128. 28737750

[ref40] Reeves L.; Wang Y.; Krauss T. F. 2D Material Microcavity Light Emitters: To Lase or Not to Lase?. Adv. Opt. Mater. 2018, 6, 1800272 10.1002/adom.201800272.

[ref41] Paik E. Y.; Zhang L.; Burg G. W.; Gogna R.; Tutuc E.; Deng H. Interlayer exciton laser of extended spatial coherence in atomically thin heterostructures. Nature 2019, 576, 80–84. 10.1038/s41586-019-1779-x. 31768043

[ref42] Liu Y.; Fang H.; Rasmita A.; Zhou Y.; Li J.; Yu T.; Xiong Q.; Zheludev N.; Liu J.; Gao W. Room temperature nanocavity laser with interlayer excitons in 2D heterostructures. Sci. Adv. 2019, 5, eaav4506 10.1126/sciadv.aav4506. 31032409 PMC6486267

[ref43] Ermolaev G.; Grudinin D.; Voronin K.; Vyshnevyy A.; Arsenin A.; Volkov V. Van Der Waals Materials for Subdiffractional Light Guidance. Photonics 2022, 9, 744 10.3390/photonics9100744.

[ref44] Zhigunov D. M.; Evlyukhin A. B.; Shalin A. S.; Zywietz U.; Chichkov B. N. Femtosecond laser printing of single Ge and SiGe nanoparticles with electric and magnetic optical resonances. ACS Photonics 2018, 5, 977–983. 10.1021/acsphotonics.7b01275.

[ref45] Prokhorov A. V.; Terekhov P. D.; Gubin M. Y.; Shesterikov A. V.; Ni X.; Tuz V. R.; Evlyukhin A. B. Resonant Light Trapping via Lattice-Induced Multipole Coupling in Symmetrical Metasurfaces. ACS Photonics 2022, 9, 3869–3875. 10.1021/acsphotonics.2c01066.

[ref46] Baranov A.; Tournié E. Semiconductor lasers. Fundamentals and applications*;* Woodhead Publishing Series in Electronic and Optical Materials; Woodhead Publishing Limited: Oxford, UK, 2013.

[ref47] Terekhov P. D.; Babicheva V. E.; Baryshnikova K. V.; Shalin A. S.; Karabchevsky A.; Evlyukhin A. B. Multipole analysis of dielectric metasurfaces composed of nonspherical nanoparticles and lattice invisibility effect. Phys. Rev. B 2019, 99, 045424 10.1103/PhysRevB.99.045424.

[ref48] Evlyukhin A. B.; Chichkov B. N. Multipole decompositions for directional light scattering. Phys. Rev. B 2019, 100, 125415 10.1103/PhysRevB.100.125415.

[ref49] Dong R.; Kuljanishvili I. Review Article: Progress in fabrication of transition metal dichalcogenides heterostructure systems. J. Vac. Sci. Technol. B 2017, 35, 030803 10.1116/1.4982736. PMC5648579 29075580

[ref50] Shi J.; Lin M.-H.; Chen I-T.; Mohammadi Estakhri N.; Zhang X.-Q.; Wang Y.; Chen H.-Y.; Chen C.-A.; Shih C.-K.; Alù A.; Li X.; Lee Y.-H.; Gwo S. Cascaded exciton energy transfer in a monolayer semiconductor lateral heterostructure assisted by surface plasmon polariton. Nat. Commun. 2017, 8, 35 10.1038/s41467-017-00048-y. 28652572 PMC5484701

[ref51] Qin M.; Xiao S.; Liu W.; Ouyang M.; Yu T.; Wang T.; Liao Q. Strong coupling between excitons and magnetic dipole quasi-bound states in the continuum in WS_2_-TiO_2_ hybrid metasurfaces. Opt. Express 2021, 29, 18026–18036. 10.1364/OE.427141. 34154071

[ref52] Yang Y.; liu W. G.; Lin Z. T.; Pan R. H.; Gu C. Z.; Li J. J. Plasmonic hybrids of two-dimensional transition metal dichalcogenides and nanoscale metals: Architectures, enhanced optical properties and devices. Mater. Today Phys. 2021, 17, 100343 10.1016/j.mtphys.2021.100343.

[ref53] Lezama I. G.; Arora A.; Ubaldini A.; Barreteau C.; Giannini E.; Potemski M.; Morpurgo A. F. Indirect-to-Direct Band Gap Crossover in Few-Layer MoTe_2_. Nano Lett. 2015, 15, 2336–2342. 10.1021/nl5045007. 25803208

[ref54] Prokhorov A. V.; Novikov S. M.; Gubin M. Y.; Shesterikov A. V.; Evdokimov P.; Putlayev V. I.; Garshev A.; Kirtaev R. V.; Zhukova E. S.; Zhukov S. S.; Miroshnichenko A. E.; Arsenin A. V.; Volkov V. S. Design and Tuning of Substrate-Fabricated Dielectric Metasurfaces Supporting Quasi-Trapped Modes in the Infrared Range. ACS Photonics 2023, 10, 1110–1118. 10.1021/acsphotonics.2c01842.

[ref55] Poleva M.; Frizyuk K.; Baryshnikova K.; Evlyukhin A.; Petrov M.; Bogdanov A. Multipolar theory of bianisotropic response of meta-atoms. Phys. Rev. B 2023, 107, L041304 10.1103/PhysRevB.107.L041304.

[ref56] Baryshnikova K. V.; Petrov M. I.; Babicheva V. E.; Belov P. A. Plasmonic and silicon spherical nanoparticle antireflective coatings. Sci. Rep. 2016, 6, 22136 10.1038/srep22136. 26926602 PMC4772069

[ref57] Spinelli P.; Verschuuren M. A.; Polman A. Broadband omnidirectional antireflection coating based on subwavelength surface Mie resonators. Nat. Commun. 2012, 3, 692 10.1038/ncomms1691. 22353722 PMC3338005

[ref58] Ruppert C.; Aslan B.; Heinz T. F. Optical Properties and Band Gap of Single- and Few-Layer MoTe_2_ Crystals. Nano Lett. 2014, 14, 6231–6236. 10.1021/nl502557g. 25302768

[ref59] Gu Q.; Fainman Y. Semiconductor Nanolasers*;* Cambridge University Press: Cambridge, UK, 2017.

[ref60] Liu C.-H.; Zheng J.; Chen Y.; Fryett T.; Majumdar A. Van der Waals materials integrated nanophotonic devices. Opt. Mater. Express 2019, 9, 384–399. 10.1364/OME.9.000384.

[ref61] Li L.; Lin M.-F.; Zhang X.; Britz A.; Krishnamoorthy A.; Ma R.; Kalia R. K.; Nakano A.; Vashishta P.; Ajayan P.; Hoffmann M. C.; Fritz D. M.; Bergmann U.; Prezhdo O. V. Phonon-Suppressed Auger Scattering of Charge Carriers in Defective Two-Dimensional Transition Metal Dichalcogenides. Nano Lett. 2019, 19, 6078–6086. 10.1021/acs.nanolett.9b02005. 31434484

[ref62] Kowalczyk H.; Biscaras J.; Pistawala N.; Harnagea L.; Singh S.; Shukla A. Gate and Temperature Driven Phase Transitions in Few-Layer MoTe_2_. ACS Nano 2023, 17, 6708–6718. 10.1021/acsnano.2c12610. 36972180

[ref63] Kim C.; Issarapanacheewin S.; Moon I.; Lee K. Y.; Ra C.; Lee S.; Yang Z.; Yoo W. J. High-Electric-Field-Induced Phase Transition and Electrical Breakdown of MoTe_2_. Adv. Electron. Mater. 2020, 6, 1900964 10.1002/aelm.201900964.

[ref64] Zhang B.; Hu C.; Xin Y.; Li Y.; Xie Y.; Xing Q.; Guo Z.; Xue Z.; Li D.; Zhang G.; Geng L.; Ke Z.; Wang C. Analysis of Low-Frequency 1/f Noise Characteristics for MoTe_2_ Ambipolar Field-Effect Transistors. Nanomaterials 2022, 12, 1325 10.3390/nano12081325. 35458035 PMC9030018

[ref65] Choi W. R.; Hong J. H.; You Y. G.; Campbell E. E. B.; Jhang S. H. Suspended MoTe_2_ field effect transistors with ionic liquid gate. Appl. Phys. Lett. 2021, 119, 223105 10.1063/5.0065568.

[ref66] Xu H.; Fathipour S.; Kinder E. W.; Seabaugh A. C.; Fullerton-Shirey S. K. Reconfigurable Ion Gating of 2H-MoTe_2_ Field-Effect Transistors Using Poly(ethylene oxide)-CsClO_4_ Solid Polymer Electrolyte. ACS Nano 2015, 9, 4900–4910. 10.1021/nn506521p. 25877681

[ref67] Ning C. Z. What is Laser Threshold?. IEEE J. Sel. Top. Quantum Electron. 2013, 19, 1503604 10.1109/JSTQE.2013.2259222.

[ref68] Li Z.-Y.; Xia Y. Metal Nanoparticles with Gain toward Single-Molecule Detection by Surface-Enhanced Raman Scattering. Nano Lett. 2010, 10, 243–249. 10.1021/nl903409x. 19958019

[ref69] Zhang Y.; Li J.; Wu Y.; Liu L.; Ming X.; Jia T.; Zhang H. Spaser Based on Dark Quadrupolar Mode of a Single Metallic Nanodisk. Plasmonics 2017, 12, 1983–1990. 10.1007/s11468-016-0471-3.

[ref70] Baek H.; Brotons-Gisbert M.; Koong Z. X.; Campbell A.; Rambach M.; Watanabe K.; Taniguchi T.; Gerardot B. D. Highly energy-tunable quantum light from moiré-trapped excitons. Sci. Adv. 2020, 6, eaba8526 10.1126/sciadv.aba8526. 32917702 PMC7486092

