# Supplementary material for: Lasing Effect in Symmetrical van der Waals Heterostructured Metasurfaces Due to Lattice-Induced Multipole Coupling
Source: Nano Lett. 2023 Nov 29;23(23):11105–11. doi: 10.1021/acs.nanolett.3c03522 (PMC10880088; doi:10.1021/acs.nanolett.3c03522)
Supplement: Supplementary file 1 — nl3c03522_si_001.pdf [file nl3c03522_si_001.pdf]

Supporting Information

Lasing effect in symmetrical van der Waals heterostructured metasurfaces due to lattice-induced multipole coupling

Alexei V. Prokhorov[1], Mikhail Yu. Gubin[1], Alexander V. Shesterikov[1], Aleksey V. Arsenin[1], Valentyn S. Volkov[1], and Andrey B. Evlyukhin[2]

[1]*Emerging Technologies Research Center, XPANCEO, Dubai 00000, United Arab Emirates*
[2]*Institute of Quantum Optics, Leibniz Universität Hannover, Hannover 30167, Germany*

S1. Algorithm for solving the problem of evaluating photoluminescence in a system with distributed feedback.

We propose a new mechanism for the realization of positive feedback using a subdiffractive resonator, which is a metasurface supporting the resonance of an octupole quasi-trapped mode (OQTM).

1. At the first stage using COMSOL Multiphysics, we optimized the size parameters and period of a metasurface composed of MoS_2/hBN/$MoTe_2$ disks and placed on SiO_2 substrate in order to match the OQTM resonance [1] and photoluminescence peak of $MoTe_2$ material at the wavelength λ_s=1146 nm [2]. The passive reflection spectrum of such a metasurface was calculated under irradiation with a linearly polarized plane wave $E_x(k_z)$ from the far-field region and has a resonant feature in the form of a peak for the reflection coefficient at the wavelength λ_s (details are discussed below).

2. At the second stage, the optical response of the metasurface with optimized parameters and irradiated from the near-field region was simulated using COMSOL Multiphysics. The near-field source is the emitting port located inside the $MoTe_2$ layer for all disks of the metasurface. The shape and area of the source correspond to the base of the disk. The detection port was located above the metasurface in the far-field region and was responsible for analyzing the superposition of waves from the source, metasurface and substrate. It is shown that the OQTM resonance is also excited by a near-field source. The quality factor of this resonance was determined, which was then used to study the lasing effect.

3. The multipole moments of the disk from the metasurface were calculated using their integral definitions [1] and the electric fields inside them obtained by means of COMSOL Multiphysics. For a qualitative analysis of the multipole contributions to the reflection (radiation) from the metasurface, the multipole expansion of the reflection coefficient under conditions of a homogeneous environment was used (details are discussed below).

4. The laser generation effect with allowance for photoluminescence from $MoTe_2$ layers under the action of external pumping at a wavelength substantially detuned from the wavelength of the OQTM resonance was revealed using the system of rate equations [3] for the carrier density and photon density of the signal (response) field at a given wavelength in the active medium. The photon density of the signal (response) field as a function of the pump intensity, the threshold conditions for laser generation, and the effective permittivity of $MoTe_2$ material under the presence of a resonator (metasurface) were obtained from the analytical stationary solutions of the rate equations.

5. The obtained effective permittivity of $MoTe_2$ material under the amplification condition was used to simulate the optical response of the metasurface (utilizing COMSOL Multiphysics facilities) in the case when emitting port with permanent intensity is located in the $MoTe_2$ amplifying medium. In the absence of other sources, we consider the signal registered by detecting port as photoluminescence from the metasurface. Under these conditions, the multipole analysis of the metasurface radiation provides information on the multipole contribution to the lasing effect.

S2. Relative permittivities of vdW materials and simulation features of few-layer flakes.

Dispersion characteristics of components of the tensor of the relative permittivity ($\varepsilon' + i\varepsilon'' = \bar{n}^2 = (n + i\alpha)^2$ for MoS_2 [4], $MoTe_2$ [5] material and real part of SiO_2 permittivity are shown in Fig.S1.

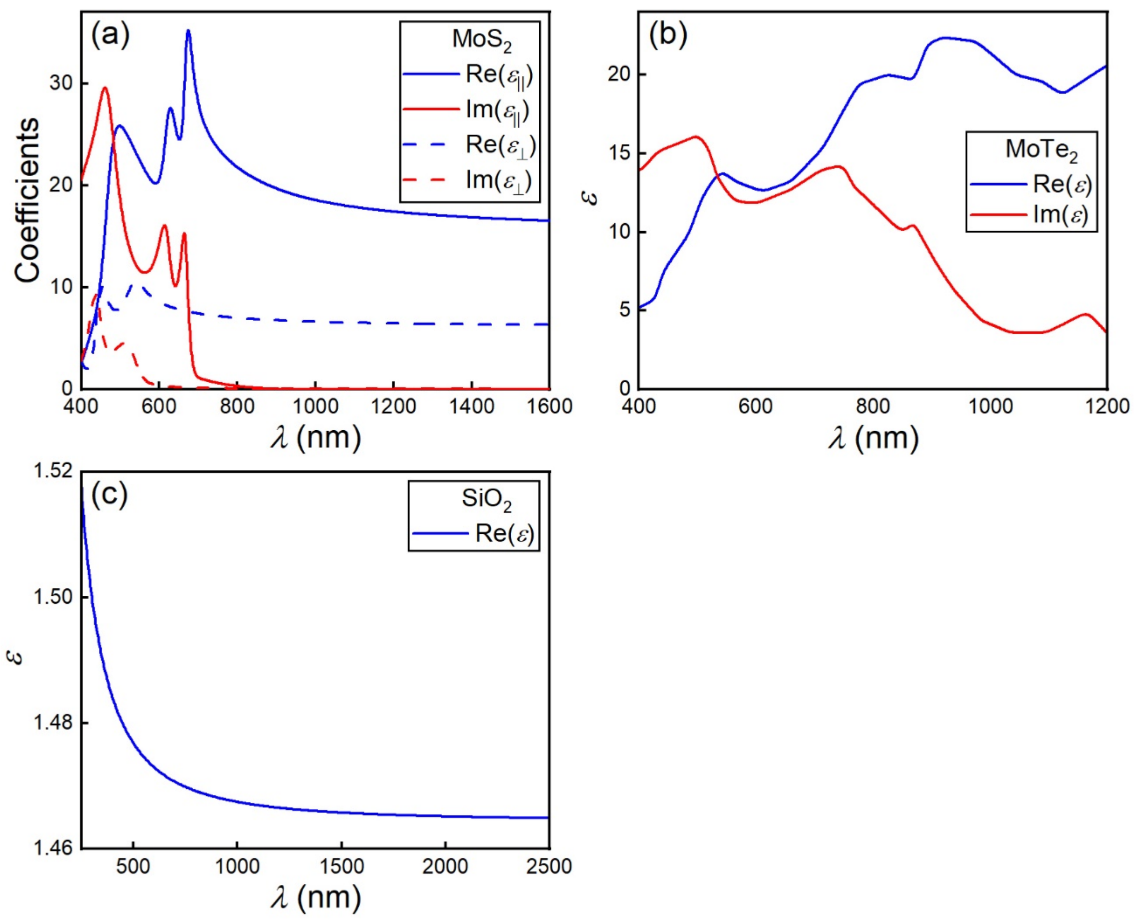

Fig. S1. Spectral dependencies for real ($\mathrm{Re}(\varepsilon) \equiv \varepsilon'$) and imaginary ($\mathrm{Im}(\varepsilon) \equiv \varepsilon''$) parts of relative permittivities for (a) MoS_2 material ($\varepsilon_{\parallel}(\varepsilon_{\perp})$ corresponds to the parallel(orthogonal) orientation of the electric field relative to the MoS_2 layers), (b) $MoTe_2$ flake, and (c) SiO_2 material.

For the numerical simulation of individual thin few-layer flakes with a real thickness Δ beyond the resolution of the computational algorithms, we calculate the relative permittivity $\varepsilon_{\mathrm{num}}(\omega)$ for numerical simulation based on the true relative permittivity of the material $\varepsilon(\omega) \equiv \varepsilon_{\mathrm{eff}}(\omega)$, but with the increased thickness Δ_{num} of the flake. We assume that relative permittivity of thin film can be described by the formula $\varepsilon = 1 + i\frac{\sigma}{\omega\Delta\varepsilon_0}$ for the true thickness Δ of the material and $\varepsilon_{\mathrm{num}} = 1 + i\frac{\sigma}{\omega\Delta_{\mathrm{num}}\varepsilon_0}$ for the increased thickness Δ_{num} of the material, where σ is the surface conductivity of the material. Using these two expressions, the effective relative permittivity can be written in the following form:

$$\varepsilon_{\mathrm{num}}(\omega) = 1 + (\varepsilon(\omega) - 1)\frac{\Delta}{\Delta_{\mathrm{num}}}, \quad \text{(S1)}$$

where Δ is the true thickness of $MoTe_2$ film, Δ_{num} is the increased thickness of $MoTe_2$ film used in numerical simulation. In our simulations, we use the two-layer $MoTe_2$ material with $\Delta = 1.4$ nm and $\Delta_{\mathrm{num}} = 20$ nm. In simulation of MoS_2/hBN/$MoTe_2$ disks, we used the adaptive spacing of spatial grid.

S3. Tuning of multipole coupling in metasurface based on MoS_2/hBN/$MoTe_2$ heterostructures and placed in air.

We start by studying the resonant properties of a single MoS_2/hBN/$MoTe_2$ disk, as well as the metasurface fabricated from such disks, under their excitation from the far-field region. The range of parameters for an isolated MoS_2/hBN/$MoTe_2$ disk is chosen so that a bright solitary octupole resonance is excited in such a disk upon lateral irradiation by the wave $E_x(k_y)$, which corresponds to the narrow region in Fig. S2a. For example, the octupole resonance arises at the wavelength λ_{oct} =1140 nm for the single disk with height H=233 nm and radius R=300 nm, see Fig. S2a. Under normal irradiation of such a disk by wave $E_x(k_z)$, there is no octupole resonance, but one can observe the excitation of a broadband electric dipole resonance [1] that dominates in this spectral range. At the same time, by optimizing the

period of a metasurface composed of such disks, it is possible to achieve the effect of lattice-induced multipole coupling [1]. Figure S2b shows the dependencies for parameters $ED_x=|Kp_x|^2$, $MD_y = |Km_y/v_d|^2$, $EQ_{xz} = |KQ_{xz}ik_d/6|^2$, and $MQ_{yz} = |KM_{yz}ik_d/(2v_d)|^2$. These dependencies allow one to estimate the contribution of nonzero components of multipoles of various orders to the total light reflection coefficient R of the metasurface, normally irradiated by the linearly-polarized plane wave $E_x(k_z)$ from the far-field region.

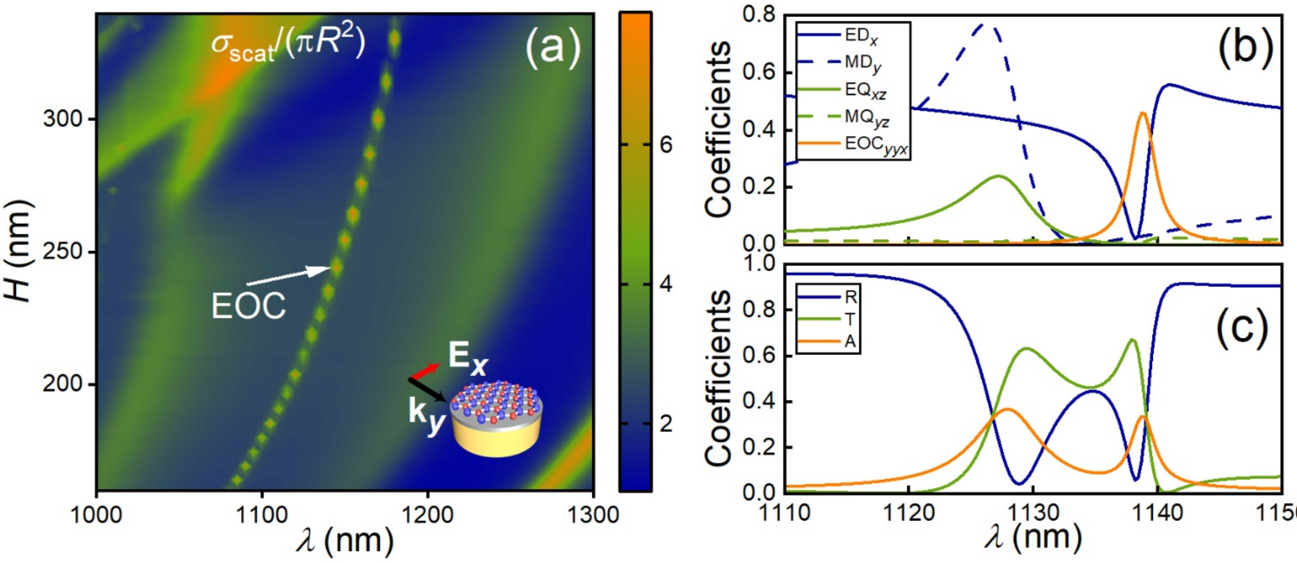

Fig. S2. (a) λ–H map of the scattering cross section of single MoS_2/hBN/$MoTe_2$ disk (material layers oriented parallel to the *xy* plane), normalized to the area of its base, with radius R=300 nm placed in air, which is irradiated by the wave $E_x(k_y)$. The dotted curve, crossing the plotting area from top to bottom, corresponds to the narrow electric octupole (EOC) resonance. (b) Spectra of the absolute values of the multipole contributions ED_x, MD_y, EQ_{xz}, MQ_{yz} to the reflection coefficient, as well as the value EOC_{yyx} corresponding to the nonradiating EOC moment of the MoS_2/hBN/$MoTe_2$ disk with height H=233 nm from the metasurface with period P=760 nm placed in air and irradiated by wave $E_x(k_z)$ from the far-field region. (c) Reflection (R), transmission (T), and absorption (A) coefficients for the metasurface as for panel (b).

Here $p_x(m_y)$ corresponds to the electric(magnetic) dipole moment and $Q_{xz}(M_{yz})$ corresponds to the electric(magnetic) quadrupole moment, respectively. In the simple case, when the metasurface is located in the air, its reflection coefficient can be described by the following expression [6]:

$$R \cong \left|K\left(p_x - \frac{m_y}{v_d} + \frac{ik_d}{6}Q_{xz} - \frac{ik_d}{2v_d}M_{yz}\right)\right|^2, \quad \text{(S2)}$$

where $K = ik_d/(E_x^0 2S_L\varepsilon_0\varepsilon_d)$, $S_L = P^2$ is the area of a lattice unit cell, P is the lattice period, $k_d = k_0\sqrt{\varepsilon_d}$ and $v_d = 1/\sqrt{\varepsilon_0\mu_0\varepsilon_d}$ are the wave number and speed of light in the surrounding medium with relative permittivity ε_d, respectively, k_0 is the wave number in vacuum, E_x^0 is the electric field of the incident (or emitted) wave in the metasurface plane, ε_0 is the vacuum permittivity, and μ_0 is the vacuum permeability. Figure S2b additionally shows the value $EOC_{yyx} = |KO_{yyx}k_d^2/6|^2$ corresponding to the resonant component O_{yyx} of the electric octupole moment (EOC), which does not give any contribution to the reflection coefficient and corresponds to the excitation of octupole eigenstates in each disk, see Fig. S2a. Note that, in our case of metasurface placed on substrate, the equation (S2) is not exactly satisfied. However, since the same scattered fields are used to calculate multipole moments of various orders under conditions of an inhomogeneous environment the hierarchy of terms in (S2) is kept in this case as well. This allows us to qualitatively estimate their contributions to the total reflection coefficient R.

The calculation of multipole components was fulfilled using semi-analytical approach based on formulas that can be found elsewhere [7]. For example, electric and magnetic dipole moments can be written in the following form [7]:

$$\mathbf{p} = \int_V \frac{\mathbf{J}(\mathbf{r}) j_0(k_d \mathbf{r})}{-i\omega} d\mathbf{r} - \frac{k_d^2}{10 i\omega} \int_V \frac{15 j_2(k_d \mathbf{r})}{(k_d \mathbf{r})^2} \left\{ [\mathbf{r} \cdot \mathbf{J}(\mathbf{r})]\mathbf{r} - \frac{1}{3} r^2 \mathbf{J}(\mathbf{r}) \right\} d\mathbf{r}, \quad \text{(S3a)}$$

$$\mathbf{m} = \frac{3}{2} \int_V \frac{[\mathbf{r} \times \mathbf{J}(\mathbf{r})] \cdot j_1(k_d \mathbf{r})}{k_d \mathbf{r}} d\mathbf{r}, \quad \text{(S3b)}$$

where V is the volume of the scatterer, $\mathbf{r}$ is the radius vector of the unit volume inside the scatterer with the absolute value $r = |\mathbf{r}| = \sqrt{x^2 + y^2 + z^2}$, $j_j(\rho) = \sqrt{\frac{\pi}{2\rho}} J_{j+\frac{1}{2}}(\rho)$ is the spherical Bessel function of i-th order. The components of the displacement current tensor in (S3) have the form:

$$\mathbf{J}(\boldsymbol{r}) = -i\omega \mathbf{P}(\mathbf{r}) = -i\omega \varepsilon_0 (\varepsilon_p - \varepsilon_d) \mathbf{E}(\mathbf{r}), \quad \text{(S4)}$$

where ω is the angular frequency of the incident (corresponds to Figs. 1b and S2b) or emitted (corresponds to Figs. 2c and S3b) field, $\mathbf{P}(\mathbf{r})$ is the polarization vector of the unit volume, ε_p is the relative permittivity of the scatterer, $\mathbf{E}(\mathbf{r})$ is the total electric field inside the particle scattered (corresponds to Figs. 1b and S2b) or emitted (corresponds to Figs. 2c and S3b) by it. In particular, the components p_x and m_y of electric and magnetic dipoles, respectively, can be written as follows:

$$p_x = \int_V \frac{J_x j_0\left(k_d \sqrt{x^2+y^2+z^2}\right)}{-i\omega} d\mathbf{r} - \frac{k_d^2}{2i\omega} \int_V \frac{\left(3x(xJ_x + yJ_y + zJ_z) - J_x(x^2+y^2+z^2)\right) j_2\left(k_d \sqrt{x^2+y^2+z^2}\right)}{\left(k_d \sqrt{x^2+y^2+z^2}\right)^2} d\mathbf{r}, \quad \text{(S5a)}$$

$$m_y = \frac{3}{2} \int_V \frac{(zJ_x - xJ_z) j_1\left(k_d \sqrt{x^2+y^2+z^2}\right)}{k_d \sqrt{x^2+y^2+z^2}} d\mathbf{r}. \quad \text{(S5b)}$$

We numerically calculate the total electric field inside particle using facilities of COMSOL Multiphysics. Next, this field is used to evaluate the dipole moments according to formulas (S3)–(S5). Thus, all other multipoles can be calculated in similar way on the basis of considered semi-analytical approach.

A characteristic feature of the multipole coupling effect in the lattice in Fig. S2b is the effective suppression of the electric dipole p_x in the vicinity of the electric octupole resonance. This occurs due to the destructive interference between the incident wave and wave scattered by the octupole, so that the local field in the metasurface plane is almost vanishes and the dipole does not radiate. For the metasurface with period P=760 nm composed of disks with H=233 nm and R=300 nm and placed in air the dipole-octupole coupling and octupole quasi-trapped mode (OQTM) formation is observed at the wavelength λ_v =1138 nm. Thus, the wavelengths of octupole resonance for the single disk and the lattice-induced OQTM resonance almost coincide, and the lattice period determines only the quality factor and the amplitude of the resonance [1]. Since the OQTM resonance is associated with the excitation of solely nonradiating components O_{yxy} (O_{yyx}, O_{xyy}), it only accumulates the energy of the near field in the metasurface plane. As a result, we obtain the lattice-induced transparency in the form of a high quality factor resonance of the metasurface's transmission coefficient at the wavelength of octupole resonance, see Fig. S2c. Note that the difference between the dependencies shown in Fig. S2 calculated for MoS_2/hBN/$MoTe_2$ disks and dependencies for monolithic MoS_2 disks are small. At the same time, the reflection dip at the wavelength 1138 nm for the MoS_2/hBN/$MoTe_2$ metasurface placed in air, see Fig. S2c, transforms into the narrow peak at the wavelength 1146 nm for the metasurface placed on SiO_2 substrate, see Fig. S3a.

S4. Resonant properties of MoS_2/hBN/$MoTe_2$ metasurface placed on SiO_2 substrate and excited from far/near-field region.

Excitation of the MoS_2/hBN/$MoTe_2$ metasurface from the near-field region, namely, by distributed near-field sources coinciding with the top base of the disks (as for lasing with external pumping at another wavelength), leads to additional spectrum narrowing of the corresponding resonance with very small shift of its central wavelength, see Fig. S3a. In this case, the near-field excitation does not violate the dipole-octupole coupling and OQTM formation near the wavelength 1146 nm, see Fig. S3b, and leads to the resonance excitation of components of all multipoles that are included in the expression for the reflection coefficient R, see Eq. (S2). Here, by the resonator spectral form factor we mean the spectrum of the total radiation normalized to the radiation of the source (emitted by the emitting port and scattered in the system without amplification) and registered by the detecting port. Thus, near-field excitation makes it possible to observe the solitary narrowband resonance associated with OQTM excitation in the metasurface and evaluate its quality factor, see Fig. S3a.

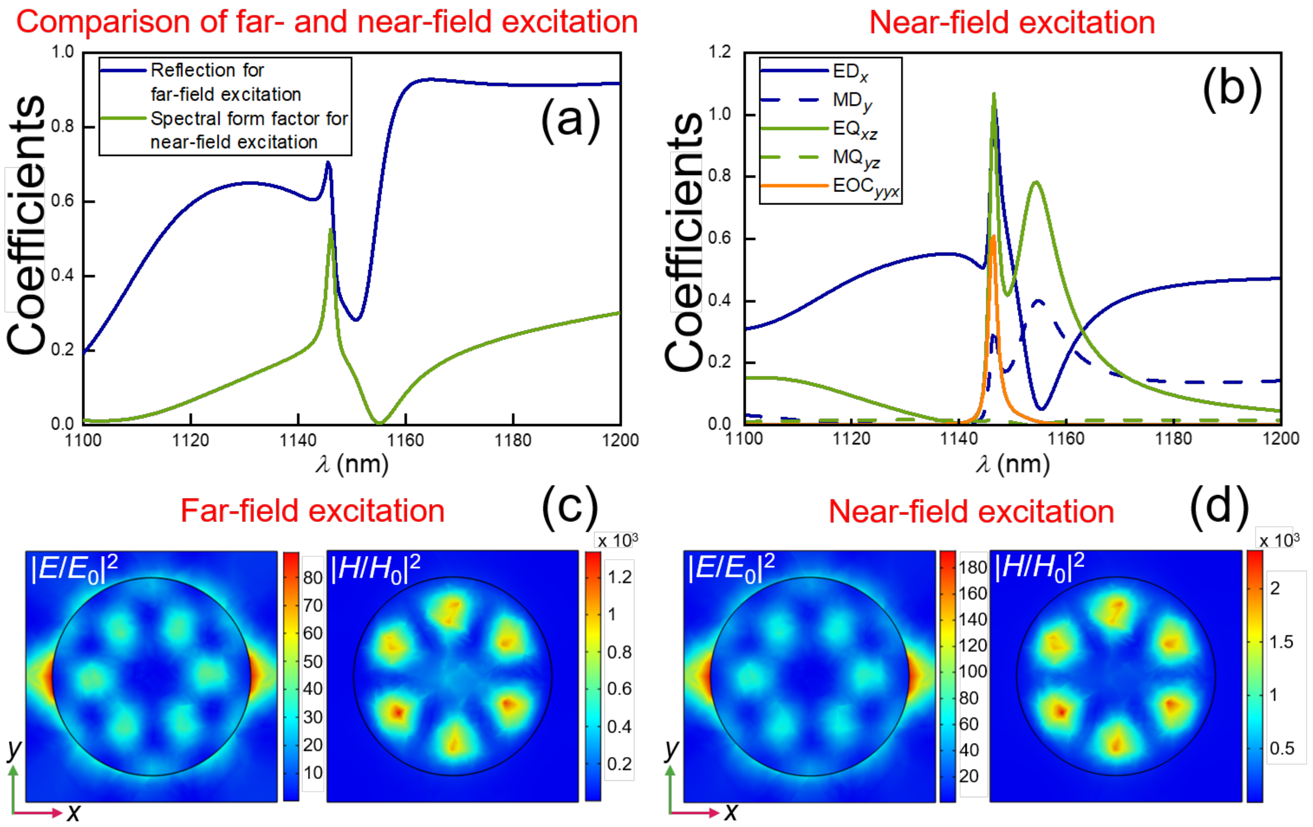

Fig. S3. (a) Comparison of reflection coefficient and resonator spectral form factor for the MoS_2/hBN/$MoTe_2$ metasurface with parameters corresponding to Fig. S2 and placed on SiO_2 substrate for different types of excitation. The OQTM resonance for the reflection coefficient appears at the wavelength 1146 nm. (b) Spectra of the absolute values of the multipole contributions ED_x, MD_y, EQ_{xz}, MQ_{yz} to the reflection coefficient, as well as the value EOC_{yyx} corresponding to the nonradiating octupole moment of the disks in the metasurface with parameters as in Fig. S2 under the near-field excitation by $E_x(k_z)$ wave. (c), (d) Visualization of the electric and magnetic fields distributions calculated for the individual building block of MoS_2/hBN/$MoTe_2$ metasurface in the horizontal cut-plane of the disk placed at height $H/2$ with parameters corresponding to Fig. S2 for (c) far-field and (d) near-field excitation at the wavelengths of reflection peaks in panel (a).

For the case of near-field excitation, the localization of electric and magnetic fields inside MoS_2/hBN/$MoTe_2$ disk is higher than for the far-filed excitation, compare Figs. S3c and S3d. Thus, the obtained solitary narrow resonance for the near-field excitation can be used as a cavity mode for the lasing regime when MoS_2/hBN/$MoTe_2$ metasurface is pumped by an external optical wave.

S5. Lasing in MoS_2/hBN/$MoTe_2$ metasurface.

S5.1. Visualization of fields and calculation of eigenmodes of MoS_2/hBN/$MoTe_2$ metasurface.

Figure S4 shows the visualization of electric and magnetic fields inside and around the pair of MoS_2/hBN/$MoTe_2$ disks from the metasurface placed on SiO_2 substrate for OQTM regime without the amplification and excited from the far-field region. The strong near-field interaction for the disks occurs in the *xz* plane for the electric field and in the *yz* plane for the magnetic field, see Figs. S4b and S4c. It can be seen that for the magnetic field, the hot spots are formed on the base of disk, see Figs. S4b and S4d, but for the electric field, the hot spots are formed at the side of the disk, see Figs. S4a and S4c. Remarkably, there is almost no interaction between disks via the magnetic field in the *xz* plane (Fig. S4a) and via the electric field in the *yz* plane (Fig. S4d).

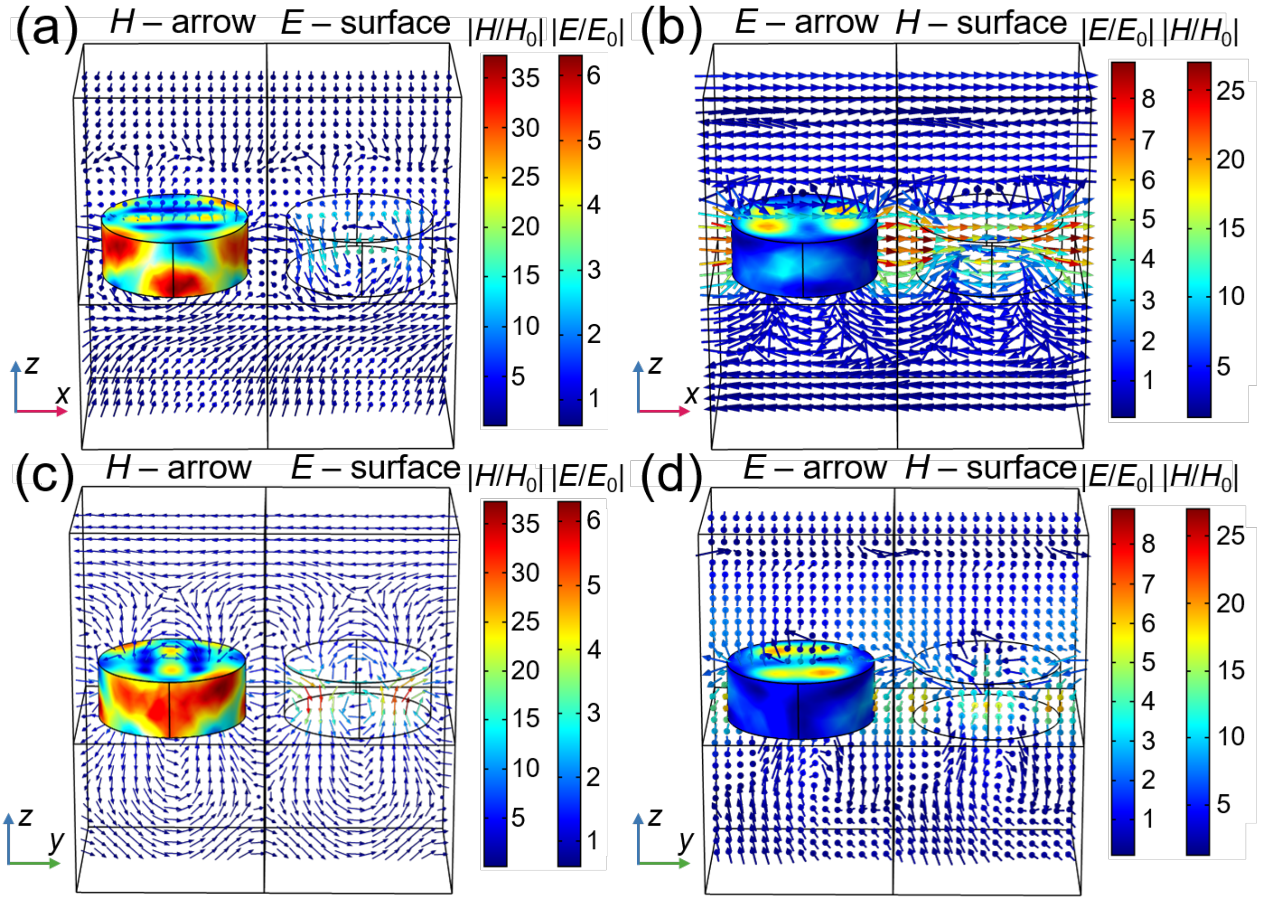

Fig. S4. The visualization of electric and magnetic field distributions calculated (a) and (b) in the *xz* plane and (c) and (d) in the *yz* plane for a pair of MoS_2/hBN/$MoTe_2$ disks from the metasurface placed on semi-infinite SiO_2 substrate and irradiated by normally incident linearly polarized wave $E_x(-k_z)$ under the conditions of the excitation of OQTM. The simulation parameters: disk's radius R=300 nm and height H=233 nm, metasurface's period P=760 nm, wavelength λ=1146 nm, $MoTe_2$ thickness Δ=1.4 nm, Δ_{num}=20 nm.

S5.2. The model of $MoTe_2$ effective relative permittivity with the pumping.

We simulated the $MoTe_2$ material under irradiation of pump field $E_x^{(p)}(k_z)$ at the wavelength λ_p assuming that the effective relative permittivity of $MoTe_2$ can be calculated as follows [8]:

$$\varepsilon_{\text{eff}}(\omega) = \varepsilon_r(\omega) + \frac{f_0\omega_0^2}{\omega_0^2-\omega^2-i\gamma\omega}, \qquad \text{(S6)}$$

where $\varepsilon_r(\omega) = \text{Re}[(\bar{n}(\omega))^2]$ corresponds to the dispersion without pumping, i.e., f_0=0; f_0 corresponds to the amplitude of amplification at the wavelength λ_0 with the Lorentzian lineshape; $\bar{n}(\omega)$ is the complex refractive index; $\gamma = 1/\tau_p$, $\tau_p = Q_0/\omega_0$ is the lifetime of the lasing mode, $\omega_0 = \frac{2\pi c}{\lambda_0}$ and Q_0 are the frequency and quality factor of the lasing mode, respectively.

We varied the value of parameter f_0 in simulations in order to increase the reflection coefficient R of the signal field from the structure, see the solid blue and dotted orange curves in Fig. 2b. In this case, we have $f_0 = \text{Im}\left[\left(n(\omega_0) + ik_g\right)^2\right]\gamma/\omega_0$ and, using the expression $k_g = -\frac{g\lambda_0}{4\pi}$ [9, 10] for the imaginary part of the refractive index, we can obtain the value of parameter g satisfying the threshold condition, see Table S2. The quality factor for the metasurface was calculated in the following form $Q_0=\lambda_0/\Delta\lambda$ using $\Delta\lambda$=FWHM for the reflection peak at the corresponding wavelength without the amplification for $MoTe_2$ material.

Note that since the considered MoS_2 material has a strong optical anisotropy, we used the relative permittivity tensor $\begin{pmatrix} \varepsilon_{\parallel}(\omega) & 0 & 0 \\ 0 & \varepsilon_{\parallel}(\omega) & 0 \\ 0 & 0 & \varepsilon_{\perp}(\omega) \end{pmatrix}$ for its description in the simulation [5].

S5.3. The operating parameters of MoS_2/hBN/$MoTe_2$ metasurface. The calculation of optical constants.

The analysis of equations (1) was carried out using the system parameters presented in Table S1. Using stationary conditions $dS/dt=dN/dt=0$ for the system of equations (1), we obtained system of algebraic equations on stationary parameters S_s and N_s. However, since its direct solutions are cumbersome we considered N_s as the varying parameter and, employing it, obtained more compact solutions of algebraic system of equations, but for the parameters $S_s(N_s)$ and $P_{\text{pump}}(N_s)$. Next, varying parameter N_s in a wide range, we calculated the photon density S_s and pump intensity $I=P_{\text{pump}}(N_s)/S_{\text{disk}}$ with parameters from Table S1 and plotted the parametric curves for dependency of S_s versus I.

The photoluminescence (reflection) curves for the lasing regime were obtained from the numerical simulation using COMSOL Multiphysics. Based on optical constants for MoS_2 material and their calculation for $MoTe_2$ material using Eq. (S6) and accounting for data in Table S1, we performed a series of numerical simulations varying the disk size and metasurface period under conditions near the generation threshold. The optimal conditions corresponding to the maximum values of the quality factor and electric field inside the disks were determined as a result of numerical optimization. For this case, the dependencies of gain coefficient and effective relative permittivity for $MoTe_2$ material on the parameters of pump beam are presented in Table S2.

Table S1. The parameters for $MoTe_2$ material.

Parameter	MoS_2/hBN/$MoTe_2$ metasurface	Other works
λ_0, nm	1146	1132 [11]
α	0.51349 [5]	
n_g	4.4242 [5]	
v_g, m/s	$6.78 \cdot 10^7$	10^8 [11]
τ_p, s	$4.10 \cdot 10^{-13}$	$1.7 \cdot 10^{-12}$ [11]
a, cm^2	$6.9 \cdot 10^{-14}$	$2.6 \cdot 10^{-15}$ [11]
τ_{sp}, s	$3 \cdot 10^{-12}$	$4 \cdot 10^{-12}$ [11]<br>$3 \cdot 10^{-12}$ [12]
τ_{nr}, s	$23 \cdot 10^{-12}$	$23 \cdot 10^{-12}$ [12]
Γ	0.03917	$1.45 \cdot 10^{-4}$ [11]
β	0.1	0.114 [11]
C, m^{6}s^{-1}	10^{-40}	10^{-40} [8]
N_{tr}, cm^{-3}	$1.61 \cdot 10^{17}$	
I_{thr}, kW/cm^2	2.94	2.1 [11]<br>(recalculated by authors of Ref. [13])
N_{thr}, cm^{-3}	$2.44 \cdot 10^{17}$	$1.318 \cdot 10^{18}$ [11]<br>10^{19} [12]

Table S2. The parameters of lasing in MoS_2/hBN/$MoTe_2$ metasurface. The pumping power recalculated for the metasurface area of about 10000 nm × 10000 nm.

№	P_{pump}, mW	I, kW/cm^2	E, kV/m	N, cm^{-3}	–g, cm^{-1}	k_g	ε_{eff}	ε_{num}	R(λ_0)	FWHM, nm (λ_0, nm)
1	0	0	0	–	–	–	$19.3096 + 4.5435i$	$2.2817 + 0.3180i$	0.53	1.7 (1146 nm)
2	1.7	1.7	113	$1.62 \cdot 10^{17}$	–40	–0.0004	$19.3096 - 0.0035i$	$2.2817 - 0.0002i$	0.84	1.48 (1146 nm)
3	2	2	123	$1.87 \cdot 10^{17}$	–1752	–0.0160	$19.3096 - 0.1416i$	$2.2817 - 0.0099i$	0.85	1.46 (1146 nm)
4	2.3	2.3	132	$2.09 \cdot 10^{17}$	–3285	–0.0300	$19.3096 - 0.2655i$	$2.2817 - 0.0186i$	0.87	1.43 (1146 nm)
5, Thr	2.94	2.94	149	$2.44 \cdot 10^{17}$	–5728	–0.0522	$19.3096 - 0.4619i$	$2.2817 - 0.0323i$	0.89	1.40 (1146 nm)
6	4	4	174	$2.71 \cdot 10^{17}$	–7550	–0.0689	$19.3096 - 0.6097i$	$2.2817 - 0.0427i$	0.91	1.37 (1146 nm)
7	16.4	16.4	351	$2.92 \cdot 10^{17}$	–9002	–0.0821	$19.3096 - 0.7264i$	$2.2817 - 0.0509i$	0.92	1.34 (1146 nm)

S5.4. Calculation of the Purcell factor.

The Purcell factor is related to the modification of radiative decay rate of the emitter placed in a cavity. Following to the Ref. [11], the Purcell factor can be calculated as follows:

$$F = F_{\max}\frac{|E_{\mathrm{MoTe_2}}|^2}{|E_{\max}|^2}\langle\cos^2\theta\rangle, \qquad \text{(S7)}$$

where $F_{\max} = \frac{3Q}{4\pi^2 V_{\mathrm{eff}}}\left(\frac{\lambda_{\mathrm{OQTM}}}{n}\right)^3$, Q is the quality factor of the resonant (OQTM) mode for the case without pumping, $V_{\mathrm{eff}} = \int \varepsilon|E|^2 dV / \max(\varepsilon|E|^2)$ is the effective mode volume, ε is the real part of dielectric permittivity of the material where electric field E is determined, λ_{OQTM} is the resonant wavelength (in our case, $\lambda_{\mathrm{OQTM}} = 1146$ nm), n=4.2 is the real part of refractive index of MoS_2 at resonant wavelength for electric field along the material layers. Since the maximum value of electric field is placed in MoS_2 disk, but not in $MoTe_2$ layer, then we use the same relations as in Ref. [11] for correct calculation of Purcell factor, i.e., in our case, $\frac{|E_{\mathrm{MoTe_2}}|^2}{|E_{\max}|^2} = 0.36$, $\langle\cos^2\theta\rangle = \frac{1}{2\pi}\int_0^{2\pi}\cos^2\theta\, d\theta = \frac{1}{2}$, θ is the angle between polarization of electric field (E_x) and direction of emission. For the considered metasurface, the Purcell factor takes the value $F = 36$. The Purcell factor determines the value of the parameter $\beta = F\beta_0$ [11]. For our system parameters, β_0 takes the value about of 0.0028. Thus, the Purcell factor is indirectly used in our calculations through the parameter β. It should be noted that, in the considered system, the Purcell factor strongly depends on the cavity mode volume, which complicates its experimental estimates [14].

S6. On the possibility of experimental fabrication of lasing metasurfaces based on quasi-trapped modes.

From the experimental point of view, the layered structures, used as the basis for creation of vdW metalasers, can be fabricated in several ways, for example, by mechanical stacking method [15] or by direct synthesis using chemical vapor deposition (CVD) method [15–17]. The mechanical stacking method utilizes exfoliation and transfer of vdW flakes followed by manual stacking them into heterostructures. The CVD method uses a “bottom-up” strategy to grow a structure from two or more TMD materials. As a result, the layered structure is obtained in the form of a stack with a given number of layers and various vdW materials. At the final stage, electron beam lithography [18] can be applied to the obtained layered structure made of vdW materials to create a metasurface with a given parameters.

References

[1] A. V. Prokhorov, P. D. Terekhov, M. Yu. Gubin, A. V. Shesterikov, X. Ni, V. R. Tuz, A. B. Evlyukhin, Resonant Light Trapping via Lattice-Induced Multipole Coupling in Symmetrical Metasurfaces, ACS Photonics, V. 9, P. 3869–3875 (2022). doi:10.1021/acsphotonics.2c01066

[2] C. Ruppert, B. Aslan, T. F. Heinz, Optical Properties and Band Gap of Single- and Few-Layer $MoTe_2$ Crystals, Nano Lett., V. 14, P. 6231–6236 (2014). doi:10.1021/nl502557g

[3] A. Baranov, E. Tournié, Semiconductor lasers. Fundamentals and applications; Woodhead Publishing Series in Electronic and Optical Materials; Woodhead Publishing Limited: Oxford, Cambridge, Philadelphia, New Delhi, 2013.

[4] G. A. Ermolaev, D. V. Grudinin, Y. V. Stebunov, K. V. Voronin, V. G. Kravets, J. Duan, A. B. Mazitov, G. I. Tselikov, A. Bylinkin, D. I. Yakubovsky, S. M. Novikov, D. G. Baranov, A. Y. Nikitin, I. A. Kruglov, T. Shegai, P. Alonso-González, A. N. Grigorenko, A. V. Arsenin, K. S. Novoselov, V. S. Volkov, Giant optical anisotropy in transition metal dichalcogenides for next-generation photonics, Nat. Commun., V. 12, P. 854 (2021). doi:10.1038/s41467-021-21139-x

[5] B. Munkhbat, P. Wrobel, T. J. Antosiewicz, T. O. Shegai, Optical constants of several multilayer transition metal dichalcogenides spectroscopic ellipsometry in the 300–1700 nm range: High index, anisotropy, and hyperbolicity, ACS Photonics V.9, P.2398 (2022). doi.org/10.1021/acsphotonics.2c00433

[6] P. D. Terekhov, V. E. Babicheva, K. V. Baryshnikova, A. S. Shalin, A. Karabchevsky, A. B. Evlyukhin, Multipole analysis of dielectric metasurfaces composed of nonspherical nanoparticles and lattice invisibility effect, Phys. Rev. B, V. 99, P. 045424 (2019). doi:10.1103/PhysRevB.99.045424

[7] A. B. Evlyukhin, B. N. Chichkov, Multipole decompositions for directional light scattering, Phys. Rev. B, V. 100, P. 125415 (2019). doi:10.1103/PhysRevB.100.125415

[8] E. Tiguntseva, K. Koshelev, A. Furasova, P. Tonkaev, V. Mikhailovskii, E. V. Ushakova, D. G. Baranov, T. Shegai, A. A. Zakhidov, Y. Kivshar, S. V. Makarov, Room-Temperature Lasing from Mie-Resonant Non-Plasmonic Nanoparticles, ACS Nano, V. 14, P. 8149–8156 (2020). doi:10.1021/acsnano.0c01468

[9] Z.-Y. Li, Y. Xia, Metal Nanoparticles with Gain toward Single-Molecule Detection by Surface-Enhanced Raman Scattering, Nano Lett., V. 10, P. 243–249 (2010). doi:10.1021/nl903409x

[10] Y. Zhang, J. Li, Y. Wu, L. Liu, X. Ming, T. Jia, H. Zhang, Spaser Based on Dark Quadrupolar Mode of a Single Metallic Nanodisk, Plasmonics, V. 12, P. 1983–1990 (2017). doi:10.1007/s11468-016-0471-3

[11] Y. Li, J. Zhang, D. Huang, H. Sun, F. Fan, J. Feng, Z. Wang, C. Z. Ning, Room-temperature continuous-wave lasing from monolayer molybdenum ditelluride integrated with a silicon nanobeam cavity, Nat. Nanotechnol., V. 12, P. 987–992 (2017). doi:10.1038/NNANO.2017.128

[12] L. Li, M.-F. Lin, X. Zhang, A. Britz, A. Krishnamoorthy, R. Ma, R. K. Kalia, A. Nakano, P. Vashishta, P. Ajayan, M. C. Hoffmann, D. M. Fritz, U. Bergmann, O. V. Prezhdo, Phonon-Suppressed Auger Scattering of Charge Carriers in Defective Two-Dimensional Transition Metal Dichalcogenides, Nano Lett., V. 19, P. 6078−6086 (2019). doi:10.1021/acs.nanolett.9b02005

[13] L. Reeves, Y. Wang, T. F. Krauss, 2D Material Microcavity Light Emitters: To Lase or Not to Lase?, Adv. Opt. Mater., V. 6, P. 1800272 (2018). doi:10.1002/adom.201800272

[14] C.-H. Liu, J. Zheng, Y. Chen, T. Fryett, A. Majumdar, Van der Waals materials integrated nanophotonic devices, Opt. Mater. Express, V. 9, P. 384–399 (2019). doi:10.1364/OME.9.000384

[15] M.-Y. Li, C.-H. Chen, Y. Shi, L.-J. Li, Heterostructures based on two-dimensional layered materials and their potential applications, Mater. Today, V. 19, P. 322–335 (2019). doi:10.1016/j.mattod.2015.11.003

[16] Z. Cai, B. Liu, X. Zou, H.-M. Cheng, Chemical Vapor Deposition Growth and Applications of Two-Dimensional Materials and Their Heterostructures, Chem. Rev., V. 118, P. 6091–6133 (2018). doi:10.1021/acs.chemrev.7b00536

[17] X. Jiang, F. Chen, S. Zhao, W. Su, Recent progress in the CVD growth of 2D vertical heterostructures based on transition-metal dichalcogenides, CrystEngComm, V. 23, P. 8239–8254 (2021). doi:10.1039/D1CE01289D
[18] Y. Yang, W. G. liu, Z. T. Lin, R. H. Pan, C. Z. Gu, J. J. Li, Plasmonic hybrids of two-dimensional transition metal dichalcogenides and nanoscale metals: Architectures, enhanced optical properties and devices, Mater. Today Phys., V. 17, P. 100343 (2021). doi:10.1016/j.mtphys.2021.100343
